# Gut Microbiota as a Molecular Regulator of Mineral Homeostasis in Horses: Mechanisms and Future Perspectives

**DOI:** 10.3390/ijms27188015

**Published:** 2026-09-09

**Authors:** Qurat Ul Ain Sajid, Muhammad Umair Asghar, Patrycja Wróblewska, Barbara Król, Mariusz Korczyński

**Affiliations:** Department of Animal Nutrition and Feed Sciences, Wroclaw University of Environmental and Life Sciences, 25 C.K. Norwida St., 51-630 Wrocław, Poland; patrycja.wroblewska@upwr.edu.pl (P.W.); barbara.krol@upwr.edu.pl (B.K.); mariusz.korczynski@upwr.edu.pl (M.K.)

**Keywords:** equine gut microbiota, mineral homeostasis, hindgut fermentation, short-chain fatty acids, intestinal mineral transporters, host–microbiota interactions, equine nutrition

## Abstract

Mineral homeostasis in horses is traditionally attributed to the coordinated regulation of the intestine, kidneys, skeleton, and endocrine system. Emerging evidence suggests the gut microbiota also shapes this process by modifying the intestinal environment where mineral absorption occurs. Despite the growing interest in host–microbiota interactions, the mechanisms linking the equine gut microbiota to mineral metabolism remain poorly understood and have not been comprehensively synthesized. This review integrates current knowledge of equine gastrointestinal physiology, gut microbial ecology, and mineral metabolism to examine how the gut microbiota influences mineral homeostasis, focusing on microbial fermentation, bioactive metabolite production, luminal pH regulation, epithelial barrier integrity, modulation of intestinal mineral transporters, and interactions with endocrine and immune pathways. The assessment of macro minerals such as calcium, phosphorus, and magnesium, along with trace minerals like iron, zinc, copper, selenium, and manganese, was conducted with a critical approach. This evaluation differentiates between results derived from equine research and those inferred from studies on other species. However, direct mechanistic evidence in horses remains limited, highlighting the need for future research. Advancing equine microbiome research through integrated microbiome profiling, metabolomics, molecular biology, and mineral balance studies will improve our understanding of host–microbiota interactions and support microbiome-informed nutritional strategies to enhance mineral utilization, gastrointestinal health, skeletal function, and long-term equine health and performance.

## 1. Introduction

Mineral homeostasis is maintained by a highly integrated physiological network that coordinates intestinal absorption, tissue distribution, endocrine regulation, and renal excretion to maintain metabolic stability despite variations in dietary mineral intake [1,2,3,4]. This regulatory network is especially intricate in horses, as nutrient absorption takes place in a uniquely specialized digestive tract evolved for hindgut fermentation [3,5,6]. Horses, being non-ruminant herbivores, depend significantly on the microbiota present in the cecum and colon to ferment structural carbohydrates that are not broken down by enzymes in the small intestine [7,8,9]. In addition to providing energy via the synthesis of short-chain fatty acids (SCFAs), this varied microbial community influences the intestinal microenvironment by modulating luminal pH, redox balance, mucus secretion, epithelial barrier stability, and the production of various bioactive metabolites [10,11,12]. Microbial functions affect the physicochemical environment for nutrient absorption while also playing an important role in immune modulation, metabolic signaling, and the preservation of intestinal equilibrium [10,13,14,15]. Thus, the horse gastrointestinal system operates as a cohesive host-microbiota ecosystem instead of simply serving as a digestive organ [7]. Despite the growing recognition of the importance of the equine gut microbiota in digestive physiology and health, its potential influence to mineral homeostasis remains poorly understood [7,16]. Investigating the interactions between microbial metabolism and mineral homeostasis may yield innovative strategies to enhance equine nutrition, health, and athletic performance. Additionally, such an understanding could guide the development of future microbiome-targeted nutritional interventions.

Mineral homeostasis has traditionally been attributed to the coordinated interactions among the intestine, skeleton, kidneys, and endocrine system, which together regulate systemic mineral balance [1,4,17]. The regulatory system oversees the metabolism of macro-minerals, including calcium (Ca), phosphorus (P), and magnesium (Mg), as well as trace minerals such as iron (Fe), zinc (Zn), copper (Cu), and selenium (Se). This system ensures that the concentrations of these minerals remain within the narrow physiological ranges necessary for normal cellular and systemic functions [2,3,18]. Within the regulatory organs, the small intestine functions as the principal site for mineral absorption. Here, the bioavailability and uptake of minerals are collectively influenced by specialized epithelial transporters, luminal physicochemical conditions, dietary composition, and nutrient interactions [19,20,21]. After intestinal absorption, minerals are allocated to metabolically active tissues, notably the skeleton. Systemic mineral balance is regulated through coordinated interactions among bone, the kidneys, and the endocrine system, involving parathyroid hormone (PTH), calcitriol [1,25-dihydroxyvitamin D_3_], fibroblast growth factor 23 (FGF23), and calcitonin [1,4,20,22].

The classical model of mineral homeostasis provides a robust framework for understanding mineral regulation. However, it inadequately addresses the influence of the intestinal microbiota, which is increasingly recognized as a crucial component in host metabolism and nutrient utilization [10,23,24]. The intestinal microbiota, in addition to supporting hindgut fermentation, operates as a dynamic ecosystem. They interact with the host by generating bioactive metabolites, modifying the intestinal environment, and affecting the epithelial, immune, and endocrine pathways associated with nutrient metabolism [10,12,25]. Interactions between host microbiota affect mineral bioavailability through several interconnected mechanisms, including the production of microbial metabolites and modulation of the intestinal microenvironment [10,12,13,14]. Evidence for these interactions is primarily derived from mechanistic studies conducted on humans and experimental animal models. These studies have demonstrated that changes in gut microbial composition and metabolic activity are associated with alterations in the metabolism of calcium, phosphorus, magnesium, iron, and zinc [26,27]. In contrast, despite the importance of hindgut fermentation in equine digestion and the increasing understanding of the equine gut microbiome, the mechanisms by which microbial communities regulate mineral homeostasis in horses remain largely unknown. This lack of knowledge impedes the advancement of nutritional strategies informed by the microbiome, which are designed to improve mineral utilization, gastrointestinal health, and athletic performance in horses [7,8,9].

Despite significant progress in understanding equine gastrointestinal physiology and the composition of hindgut microbiota, the functional relationships between microbial communities and mineral balance remain inadequately understood. Current knowledge is primarily based on studies conducted with humans and laboratory animals, whereas research involving horses remains both scarce and fragmented. Consequently, significant questions remain unresolved, including those concerning the influence of microbial metabolites on mineral bioavailability, the regulation of intestinal mineral transport systems, and the interaction between the gut microbiota and endocrine pathways that govern mineral metabolism [7,10,12]. Addressing these knowledge gaps is crucial because a more comprehensive understanding of host–microbiota interactions may facilitate the development of microbiome-based nutritional strategies. These strategies aim to improve mineral utilization, support gastrointestinal health, enhance skeletal integrity and athletic performance, and promote the long-term health and welfare of horses. Due to the limited and scattered nature of direct equine data on this topic across various disciplines, the primary contribution of this review is to integrate the scarce equine-specific evidence with mechanistic findings from other species into a cohesive framework. This approach explicitly differentiates established knowledge in horses from inferences made through extrapolation, thereby formulating testable hypotheses for future molecular research focused on equines. We propose that the equine gut microbiota potentially influences mineral homeostasis through a complex network of mechanisms rather than a singular pathway. These mechanisms may encompass the microbial transformation of dietary mineral-containing compounds, the production of microbial metabolites that alter the intestinal physicochemical environment, impacts on epithelial barrier function and mineral transport processes, and indirect interactions with host immune and endocrine pathways. Nevertheless, the extent to which these mechanisms are active in horses remains unclear, and evidence from humans and other experimental species should not be equated with direct equine evidence. Consequently, this review assesses these proposed pathways based on the strength and origin of the available evidence, differentiating experimentally verified equine mechanisms from cross-species evidence and hypotheses that require validation in horses. This review therefore integrates existing knowledge of equine gastrointestinal physiology, gut microbial ecology, and mineral homeostasis to evaluate these potential mechanisms, distinguish established equine evidence from cross-species inference, and identify priorities for future research. These efforts aim to advance microbiome-informed nutritional strategies and enhance the long-term health and welfare of equines.

## 2. Literature Search and Evidence Classification

A thorough literature review was performed utilizing the databases PubMed, Web of Science, Scopus, Science Direct, and Google Scholar to locate pertinent peer-reviewed articles concerning equine gut microbiota, mineral homeostasis, gastrointestinal physiology, host–microbiota interactions, and mineral metabolism. The search encompassed studies published up to July 2026, employing combinations of keywords including animal model (“equine”, “horse”, “horses”), microbial ecosystem (“gut microbiota”, “gut microbiome”, “hindgut fermentation”), and mineral metabolism (“mineral homeostasis”, “mineral metabolism”, “calcium”, “phosphorus”, “magnesium”, “iron”, “zinc”, “copper”, “selenium”, and “intestinal absorption”). Additional searches combined individual minerals with mechanistic terms including “microbial metabolites”, “short-chain fatty acids”, “intestinal transporter”, “bioavailability”, “phytate”, “epithelial barrier”, “endocrine regulation”, and “immune signaling”. Research articles, systematic reviews, authoritative review papers, books, and nutritional guidelines published in English were included in the analysis. Literature was prioritized when it provided direct evidence in horses; however, studies conducted in humans and other animal models were also included when they provided mechanistic evidence relevant to pathways potentially applicable to equine mineral homeostasis. Foundational studies were retained where necessary to establish physiological or molecular mechanisms. Studies published in the past 10 to 15 years were prioritized, although landmark publications and foundational references were incorporated as necessary. The chosen literature underwent critical evaluation and was organized into thematic sections that covered equine gastrointestinal physiology, gut microbial ecology, mineral homeostasis, host–microbiota interactions, molecular mechanisms, and future research directions. Studies were excluded if they did not pertain to equine gastrointestinal physiology, gut microbial function, mineral metabolism, or mechanisms pertinent to host–microbiota interactions, or if they lacked sufficient information to substantiate the mechanistic interpretation offered in this review. As this article constitutes an integrative mechanistic narrative review, rather than a systematic review or meta-analysis, studies were synthesized thematically instead of being subjected to quantitative pooling.

To prevent conflating equine observations with mechanistic evidence from other species, the evidence synthesized in this review was interpreted based on its experimental origin. Findings were considered direct equine evidence when the microbiota, microbial metabolites, mineral status, mineral absorption, transporter expression, or related physiological outcomes were experimentally evaluated in horses. Findings from humans or other animal models were classified as cross-species mechanistic evidence when they provided experimentally supported mechanisms potentially relevant to equine physiology but had not been directly demonstrated in horses. Mechanisms lacking sufficient experimental validation were described as proposed or hypothetical mechanisms. This classification was applied throughout the text and tables, and causal terminology was moderated where direct evidence was unavailable.

## 3. Equine Gastrointestinal Tract and Gut Microbiota

### 3.1. Structural and Functional Organization of Equine Gastrointestinal Tract

The equine digestive system is a specialized anatomical structure evolved to efficiently process fibrous plant diets, primarily through extensive fermentation occurring in the hindgut [28,29]. In contrast to ruminants, which primarily undergo microbial fermentation in the forestomach, horses are monogastric herbivores. They depend on a large and metabolically active cecum and colon for the breakdown of structural carbohydrates that are not enzymatically digested in the small intestine [3,6,30]. This evolutionary trait allows horses to extract energy from plant cell wall components while ensuring a steady movement of digesta through the digestive tract, a crucial feature that aligns with their natural grazing habits and nutritional ecology [5,31]. The equine hindgut functions as both a fermentation site and a dynamic ecosystem. Within this environment, complex interactions between the host and its resident microbiota influence digestive efficiency, intestinal balance, immune function, and nutrient absorption [7,8,9]. Thus, comprehending the structural and functional organization of the equine gastrointestinal tract provides the physiological foundation for appreciating the composition, metabolic activity, and ecological complexity of the equine gut microbiota.

**Figure 1:**
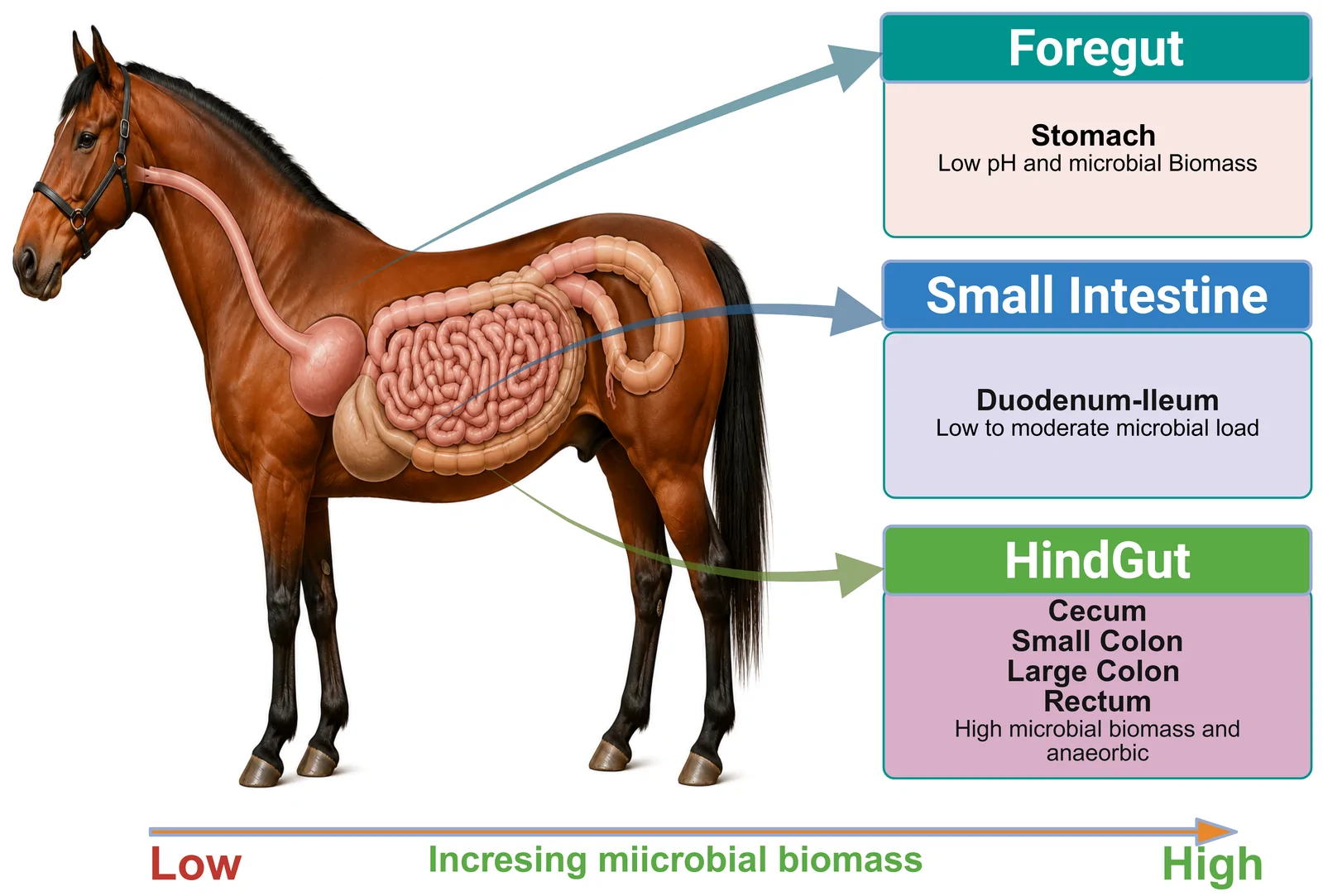
Schematic representation of the equine gastrointestinal tract. The anatomical organization of the equine gastrointestinal tract provides the structural basis for understanding regional microbial distribution, hindgut fermentation, nutrient utilization, and interactions relevant to mineral homeostasis. The color coding distinguishes the major gastrointestinal compartments, while the gradient from low to high indicates increasing microbial biomass along the gastrointestinal tract. Created by the authors using BioRender. https://BioRender.com/bek9122 (accessed on 12 August 2026).

The equine gastrointestinal system is functionally divided into regions with distinct roles in digestion and nutrient utilization. The stomach and small intestine are responsible for the enzymatic breakdown and absorption of readily digestible nutrients, whereas a substantial proportion of structural carbohydrates reaches the hindgut for microbial fermentation [3,5,6]. The cecum and colon provide the principal site of microbial fermentation, where resident microorganisms degrade cellulose, hemicellulose, and other complex polysaccharides, and generate microbial metabolites relevant to host physiology [7,30]. This anatomical organization creates a distinct microbial environment that supports extensive hindgut fermentation and sustained host–microbiota interactions [3,6]. These features are especially pertinent to mineral homeostasis, as microbial activity in the hindgut can alter the luminal chemical environment and produce metabolites that potentially affect mineral availability and host physiological pathways. The anatomical organization of the equine gastrointestinal tract and the major digestive compartments relevant to microbial colonization are illustrated in Figure 1.

The physicochemical conditions of the equine hindgut, characterized by extended digesta retention, a stable luminal environment, and a continuous influx of fermentable substrates, facilitate the sustenance of diverse anaerobic microbial communities and promote extensive fermentation [6,30]. Microbial fermentation converts structural carbohydrates into short-chain fatty acids (SCFAs), primarily acetate, propionate, and butyrate, which contribute substantially to host energy metabolism and influence epithelial and gastrointestinal function [7,10,11]. Beyond carbohydrate fermentation, hindgut microorganisms produce diverse metabolites that can interact with host physiological pathways [25,27]. Microbial activities are pertinent to mineral homeostasis as they have the capacity to alter the intestinal physicochemical environment. Additionally, they may offer microbial-derived signals that enable the microbiota to affect mineral availability and host mineral physiology.

### 3.2. Composition, Development, and Ecological Diversity of the Equine Gut Microbiota

The adult equine gut microbiota comprises a diverse and predominantly anaerobic community adapted to the fermentative conditions of the hindgut [32]. Although a relatively stable core community is maintained, its composition and metabolic activity can vary with factors such as diet, age, management, and antimicrobial exposure [7,33]. These variations are pertinent to mineral homeostasis, as dietary and environmental changes can influence microbial composition and function. Such changes may alter fermentation patterns, microbial metabolite production, and the intestinal physicochemical environment. Therefore, examining microbiota composition alone may be inadequate for comprehending its potential contribution to mineral physiology; functional activity and host–microbiota interactions are also significant [16,34,35].

The mature hindgut microbiota of horses is primarily composed of a wide array of anaerobic micro-organisms that together create a highly specialized fermentative environment. Bacteria are the dominant group within this microbial community, with the phyla *Firmicutes*, *Bacteroidota*, *Fibrobacterota*, *Verrucomicrobiota*, *Proteobacteria*, and *Actinobacteriota* playing key roles in breaking down fiber and maintaining metabolic balance [8,33,34,36,37]. The reported relative abundances of microbial phyla in the equine gut microbiota exhibit significant variability across studies. *Firmicutes* are estimated to constitute approximately 45–68%, of the gut microbiome, while *Bacteroidetes* account for 14–37% in certain reports [16]. Conversely, another study identified *Bacteroidetes* as being more prevalent than *Firmicutes* in both healthy and colitic horses [38]. *Fibrolytic* bacteria are responsible for the degradation of cellulose, hemicellulose, and other structural polysaccharides. In contrast, saccharolytic and proteolytic microorganisms further process the intermediate products of fermentation. This results in complex trophic interactions that improve the efficiency and stability of hindgut fermentation [7,30,39,40]. Besides bacteria, the equine hindgut also contains methanogenic archaea, anaerobic fungi, protozoa, bacteriophages, and other viruses. Although these are less prevalent, they play a role in the microbial ecosystem by utilizing hydrogen, disrupting plant fibers, regulating microbial populations, and facilitating horizontal gene transfer [7,36,41,42,43].

Forage-based diets encourage the growth of fibrolytic microorganisms, while diets rich in starch or concentrates promote amylolytic bacterial populations. This shift can disrupt microbial equilibrium, thereby elevating the risk of hindgut acidosis and related gastrointestinal disorders [39,40,44,45]. Various factors, including age, breed, genetics, management practices, geographical location, seasonal variations, exercise, stress, disease status, and antimicrobial usage, contribute to differences in microbial composition and function among individuals [34,35,37,46,47]. Although differences exist, the functional redundancy among microbial taxa enables the hindgut ecosystem to sustain crucial fermentative and metabolic activities. This redundancy ensures digestive efficiency and gastrointestinal balance across a wide range of physiological conditions [7,43,48]. Each taxonomic group of microbial communities contributes distinctively to metabolic processes, which assist in fiber degradation, promote microbial cross-feeding, sustain gastrointestinal equilibrium, and potentially affect mineral metabolism (Table 1).

### 3.3. Functional Roles of the Equine Gut Microbiota

The microbiota of the equine hindgut plays a crucial role in the degradation of dietary fiber that escapes enzymatic digestion in the foregut, thereby enabling horses to efficiently digest diets rich in forage. This process is facilitated by the coordinated activity of diverse fibrolytic microorganisms, which produce a broad spectrum of carbohydrate-active enzymes (CAZymes). These enzymes are capable of degrading cellulose, hemicellulose, pectin, and other plant polysaccharides into fermentable substrates [30,36,53,58,59,60]. These microorganisms participate in complex metabolic networks, wherein primary degraders generate intermediate products subsequently utilized by secondary fermenters through microbial cross-feeding. This process optimizes fiber degradation and energy extraction [11,33,36,49]. Such a collaborative metabolic strategy enables the equine hindgut to function as an efficient fermentation system, thereby enhancing digestive efficiency while preserving the metabolic activities of the resident microbiota [37,59].

During the anaerobic fermentation of dietary carbohydrates, acetate, propionate, and butyrate are generated, collectively providing an estimated 50–70% of the horse’s maintenance energy requirements. SCFAs are established products of equine hindgut fermentation and contribute substantially to host energy metabolism [7,10,11,61]. Research in rodents and humans shows that SCFA signaling can affect pathways related to the epithelium and minerals [12,13,14]. It is still unclear whether these effects are similar in horses. SCFAs are known as products of microbial fermentation, but they also act as signaling molecules that impact intestinal epithelial, immune, and metabolic functions. Studies in horses highlight their role in hindgut fermentation and gastrointestinal health, while specific effects on epithelial signaling, nutrient transport, and mineral pathways are mainly observed in humans and animal models [12,13,14]. These cross-species findings suggest biologically plausible mechanisms for interactions between equine microbiota and their hosts. However, they should not be construed as direct evidence of short-chain fatty acid (SCFA)-mediated regulation of mineral absorption in horses. The hindgut microbiota is responsible for the production of essential vitamins, including those in the vitamin B complex and vitamin K. It also participates in amino acid metabolism and nitrogen recycling while generating numerous bioactive metabolites that facilitate communication between the intestinal microbiota and host tissues [7,25,27]. Collectively, these microbial metabolites extend the functional impact of the gut microbiota beyond digestion. They establish a metabolic interface that links microbial activity with host physiological regulation, thereby providing a mechanistic basis for the emerging role of the gut microbiota in mineral homeostasis [37].

**Figure 2:**
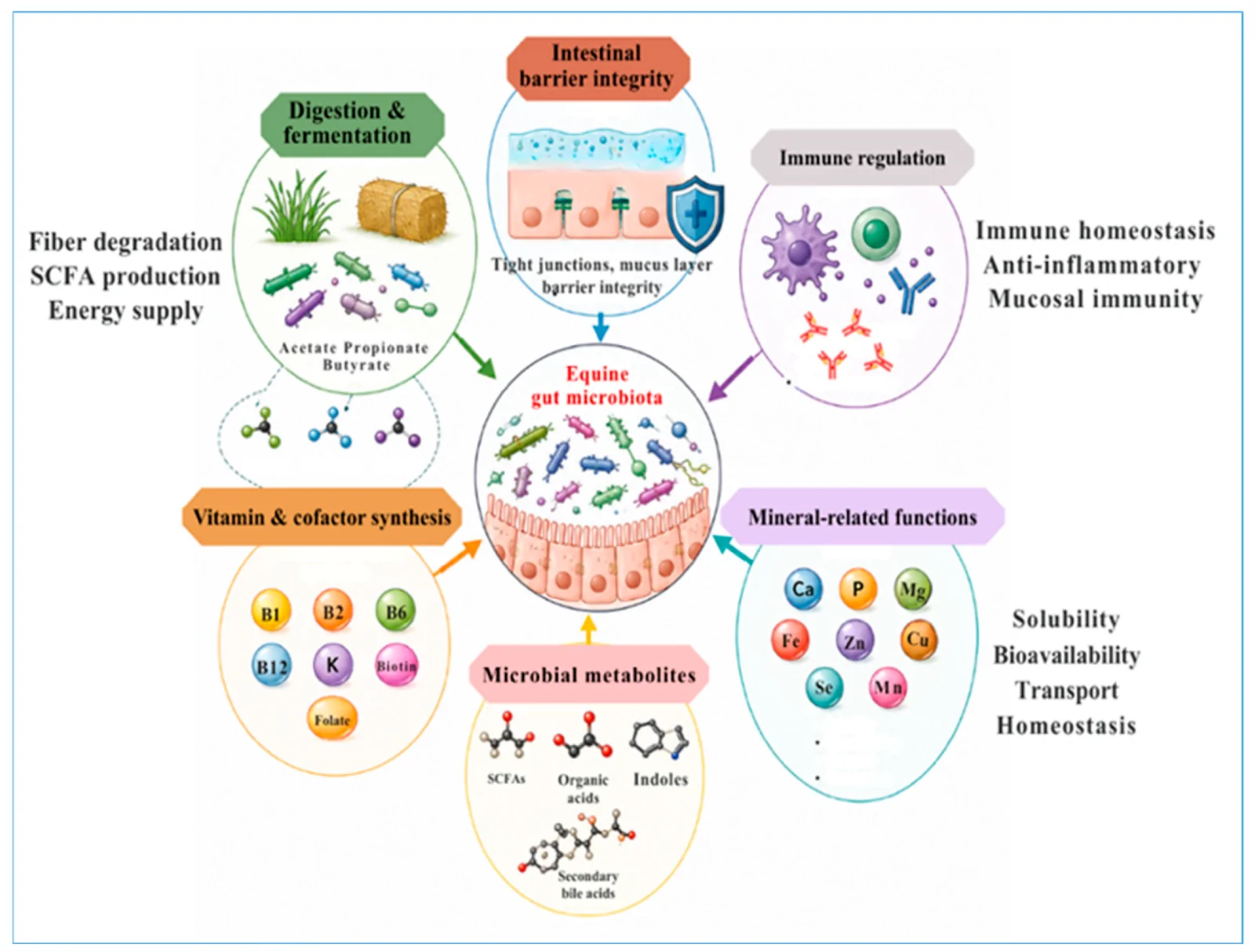
Major functional roles of the equine gut microbiota. The equine gut microbiota contributes to fiber degradation and fermentation, short-chain fatty acid production, intestinal barrier integrity, immune regulation, vitamin and cofactor synthesis, microbial metabolite production, and mineral-related functions, thereby supporting gastrointestinal and systemic physiological homeostasis. Created by authors using BioRender. https://BioRender.com/wme4zom (accessed on 12 August 2026).

Beyond its involvement in energy metabolism, the gut microbiota in horses plays a vital role in maintaining intestinal equilibrium. The metabolites generated by these microorganisms fortify the intestinal epithelial barrier by preserving the integrity of tight junctions and promoting mucus secretion. Consequently, this reduces intestinal permeability, thereby providing protection against gut pathogens and inflammatory stimuli [12,13]. The gut microbiota exerts an influence on both innate and adaptive immune responses through continuous interactions with epithelial and immune cells. Microbe-derived metabolites, particularly butyrate, facilitate the differentiation of regulatory T cells and reduce excessive inflammatory reactions, thereby promoting immune tolerance within the intestines [10,14,15,37]. Microbial communities provide an essential mechanism of colonization resistance, which improves gastrointestinal health and maintains microbial balance [62,63]. These functions collectively establish the gut microbiota as a crucial regulator of intestinal integrity and overall host health, rather than merely a participant in feed fermentation [64]. The principal physiological functions of the equine gut microbiota and their contributions to host metabolism and mineral homeostasis are summarized in Figure 2.

### 3.4. Studying the Equine Enteral Microbiome: Techniques to Characterize Microbial Communities

**Figure 3:**
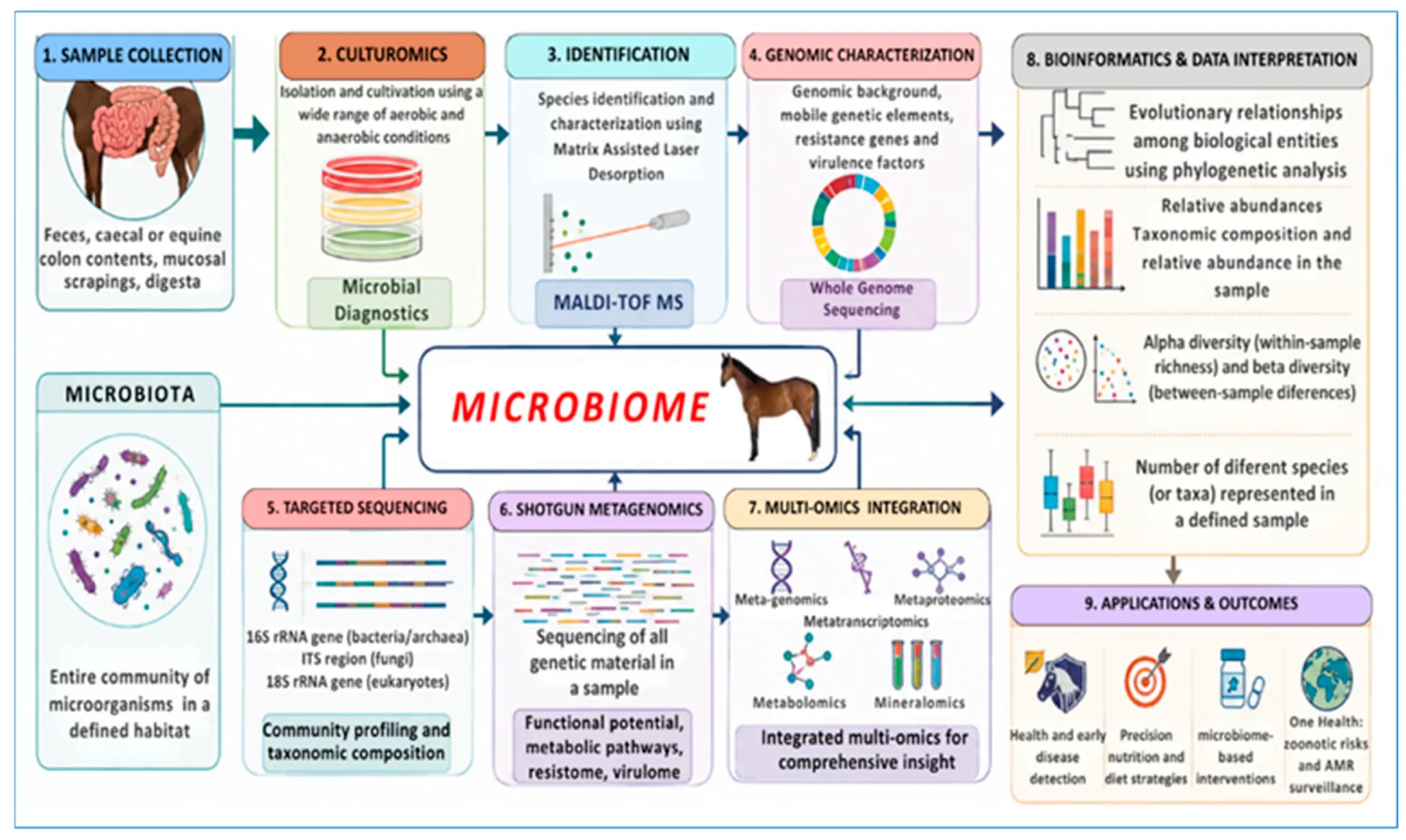
Overview of methodological approaches for characterizing the equine gut microbiome. The workflow integrates culture-dependent approaches, including culturomics, MALDI-TOF mass spectrometry, and whole-genome sequencing, with molecular and sequencing-based approaches, including 16S rRNA, ITS, and 18S rRNA amplicon sequencing and shotgun metagenomics. Integration with meta-transcriptomic, met proteomics, metabolomics, and mineral omics enables the functional characterization of microbial communities and host–microbiota interactions. Created by authors using BioRender. https://BioRender.com/nz99hnd (accessed on 12 August 2026).

The characterization of the equine gut microbiota has experienced a significant methodological evolution over the past two decades. Initially, studies predominantly employed culture-dependent techniques, which were limited to identifying only those bacterial taxa that could proliferate under standard laboratory conditions, thereby significantly underestimating the actual microbial diversity [65]. Recent advancements in culturomics, which integrate diverse culture conditions with rapid identification through matrix-assisted laser desorption/ionization time-of-flight mass spectrometry, have partially mitigated this limitation [66,67]. However, the field has been fundamentally transformed by the introduction of next-generation sequencing technologies [68,69]. Figure 3 provides a summary of the primary culture-based and sequencing-based methodologies employed to characterize the equine gut microbiome, highlighting their convergence on common downstream bioinformatics analyses.

The predominant sequencing method involves targeting the bacterial 16S ribosomal RNA gene, wherein its hypervariable regions are amplified, clustered into operational taxonomic units, and subsequently classified using reference databases such as Green-genes DeSantis et al. [70] and SILVA Quast et al. [71]. Although this 16S amplicon sequencing is both cost-effective and well-established, it presents certain limitations in equine microbiome research. Specifically, current short-read platforms generally capture only a portion of the complete gene, which limits the ability to confidently assign taxonomy beyond the genus level [72]. Additionally, variations in 16S copy number across different taxa can skew estimates of relative abundance [73,74], and the selection of primers may systematically over- or under-represent specific bacterial groups [69,75]. Targeted Enrichment Hybrid Capture (TEHC) represents an alternative sequencing method that has been investigated for characterizing the equine fecal microbiome. This approach has been shown to identify a greater diversity of genera and species compared to conventional 16S rRNA amplicon sequencing. Notably, TEHC facilitates the detection of additional phyla, such as *Tenericutes* and *Euryarchaeota* [76]. These findings suggest that TEHC may provide a more comprehensive option for future studies on the equine microbiome.

Shotgun metagenomics sequencing addresses several limitations by capturing the complete gene content of a sample, rather than focusing on a single marker gene. This approach enhances taxonomic resolution, and crucially for the questions explored in this review, allows for the direct assessment of functional gene content. This includes genes that may be involved in mineral transport, sequestration, or metabolite production [77]. Although this approach is computationally intensive, its interpretive capacity is limited by the availability of reference genomes. Non-pathogenic and previously uncultured taxa are comparatively underrepresented in public sequence databases, a deficiency frequently referred to as microbial “dark matter” [69,77,78]. Bioinformatics platforms commonly employed for the analysis of 16S and metagenomic data, such as QIIME [79] and mothur Schloss et al. [80], facilitate the computation of diversity indices. These indices include the Shannon index [81], Simpson index [82], and the Chao1 richness estimator [83], which characterize community richness and evenness both within and between samples. Future research on equine mineral homeostasis, which involves integrating shotgun metagenomics with metabolomic and mineral-balance data as detailed in Section 6, will require the ongoing development of equine-specific reference databases to fully leverage these methodological advancements [69].

## 4. Physiological Regulation of Mineral Homeostasis in Horses

### 4.1. Physiological Importance of Minerals

Minerals are crucial nutrients involved in nearly all physiological processes necessary for growth, maintenance, reproduction, and athletic performance in horses. Macro minerals like Ca, P, and Mg are needed in substantial quantities and are vital for skeletal development, neuromuscular function, energy metabolism, and maintaining acid–base balance [2,84]. Trace minerals, including Fe, Zn, Cu, Mn, and Se, are required in minimal quantities; however, they are equally essential. These minerals function as structural components or enzyme cofactors, playing critical roles in oxygen transport, antioxidant defense, immune regulation, connective tissue formation, and cellular metabolism [2,3,17]. Mineral levels are meticulously regulated through mechanisms that govern intestinal absorption, tissue storage, redistribution, and excretion, all in accordance with metabolic requirements. This regulation is crucial because both deficiencies and excesses can impair physiological functions [1,4,85].

Maintaining an appropriate mineral balance is essential, as both deficiencies and excesses can adversely affect equine health, growth, reproduction, immune function, and athletic performance. Mineral imbalances may arise from inadequate dietary intake, diminished intestinal absorption, excessive excretion, or antagonistic interactions among minerals that reduce their bioavailability. For example, an excessive dietary intake of Ca can inhibit P absorption, whereas elevated Fe levels can obstruct the absorption of Cu and Zn. The deficiency of Se, Cu, or Zn has been associated with decreased antioxidant capacity, impaired immune response, delayed tissue healing, and reduced musculoskeletal performance [2,3,17]. Consequently, achieving mineral balance necessitates not only adequate dietary intake, but also efficient intestinal absorption, physiological regulation, and the synergistic interaction of various minerals to support optimal metabolic processes. The major minerals required for normal physiological function are regulated through highly coordinated homeostatic mechanisms. Emerging evidence suggests that several of these regulatory pathways may also be influenced by the gut microbiota, as summarized in Table 2.

### 4.2. Intestinal Absorption and Transport of Minerals

In equines, mineral absorption predominantly occurs in the small intestine, although the large intestine also contributes to the absorption of specific minerals and electrolytes [94,95]. This absorption process is precisely regulated and is affected by various factors, including the physicochemical properties of each mineral, intestinal pH levels, dietary composition, and the presence of specific epithelial transport proteins [19,20,21,95]. Minerals are absorbed via both passive paracellular diffusion and active transcellular transport, with the significance of each pathway changing based on mineral concentration, physiological needs, and hormonal influences [94,95]. These processes collectively ensure that mineral absorption corresponds with the horse’s metabolic requirements, thereby preventing excessive accumulation.

Mineral absorption within the body is mediated by specific membrane transporters situated in the intestinal epithelial cells. These transport mechanisms regulate the intake, intracellular movement, and release of minerals according to the body’s physiological requirements. The absorption of calcium predominantly involves transient receptor potential vanilloid type 6 (TRPV6), calbindin-D9k, and plasma membrane calcium ATPase (PMCA1b) [94,95]. Phosphorus transport is primarily carried out by sodium-dependent phosphate cotransporters-NaPi-IIb (SLC34A2) [96], while magnesium absorption relies heavily on transient receptor potential melastatin (TRPM) channels. Fe is absorbed via divalent metal transporter 1 (DMT1) and ferroportin. The regulation of Zn and Cu levels involves the ZIP, ZnT, ATP7A, and ATP7B transporter families [20,21,87,89]. The expression and function of these transporters are constantly modified in response to dietary mineral intake, hormonal signals, and physiological needs. This adaptive regulation ensures effective mineral absorption, prevents excessive accumulation, and maintains overall mineral balance in the body. The presence and physiological function of these transport systems must be differentiated from evidence indicating their regulation by the gut microbiota. Direct evidence for the microbiota-mediated regulation of mineral transporter expression or activity in horses is currently limited. The proposed interactions between microbiota and transporters, which are discussed below, are primarily supported by experimental studies conducted in humans or other animal models.

Mineral absorption is influenced by various dietary and physiological factors, as well as transporter activity. The solubility and bioavailability of minerals within the intestines are contingent upon their chemical form. Dietary components can either facilitate or impede absorption through chemical interactions within the intestinal lumen. For example, phytate, oxalate, and elevated dietary fiber levels reduce the bioavailability of minerals by forming insoluble complexes with Ca, Fe, Zn, and other trace elements. In contrast, organic acids and fermentation products increase mineral solubility and facilitate absorption [26,97]. Factors such as gastrointestinal pH, intestinal transit time, and the integrity of the epithelium also affect mineral uptake by altering the physicochemical conditions at the absorption site [19,20]. Effective mineral absorption is contingent upon both dietary intake and the maintenance of a healthy intestinal environment. This understanding constitutes the physiological foundation for the potential influence of gut microbiota on mineral homeostasis.

The spatial relationship between hindgut microbiota and mineral absorption is a critical factor in understanding microbiota–mineral interactions in horses. A key aspect to consider is the spatial separation between the primary site of microbial fermentation and the main sites of mineral absorption. The majority of microbial biomass and metabolic activity is located in the cecum and colon, whereas significant absorption of various minerals takes place in the small intestine. Therefore, it should not be presumed that hindgut microorganisms directly enhance mineral absorption at the site of microbial fermentation. Instead, their impact may be mediated through both local and indirect mechanisms. Locally, microbial metabolism can modify luminal pH, mineral speciation, complexation, and the chemical environment of the hindgut, which may influence the availability or recycling of minerals within this compartment. Indirectly, microbial metabolites produced in the hindgut may interact with the epithelial, immune, neural, or endocrine pathways, thereby affecting physiological processes elsewhere in the gastrointestinal tract or systemically. The relative significance of these pathways in horses remains uncertain and necessitates direct experimental investigation.

### 4.3. Endocrine Regulation of Mineral Homeostasis

Mineral homeostasis is maintained through a precisely coordinated endocrine system that continuously adjusts intestinal absorption, renal reabsorption, and bone mineral exchange to fulfill physiological requirements. The primary hormones regulating this system are the parathyroid hormone (PTH), calcitriol (1,25-dihydroxyvitamin D_3_), fibroblast growth factor 23 (FGF23), and calcitonin. These hormones quickly react to shifts in circulating mineral levels and operate through complementary feedback loops to maintain overall mineral equilibrium [1,4,20,85]. Their coordinated actions maintain mineral levels within stringent physiological limits, despite fluctuations in dietary intake and metabolic demands [1].

PTH is secreted in response to reduced blood calcium levels. It increases renal Ca reabsorption, stimulates the release of bone minerals, and promotes the synthesis of calcitriol in the kidneys. Calcitriol subsequently enhances the intestinal absorption of Ca and P by upregulating the expression of mineral transport proteins in enterocytes [1,20]. FGF23 is predominantly produced by osteocytes and osteoblasts in response to increased P concentrations. Its role is to decrease renal phosphate reabsorption, inhibit the synthesis of calcitriol, and promote phosphate excretion, thus ensuring P homeostasis [1,22]. Although calcitonin has a more restricted role in adult horses, it contributes to Ca regulation by diminishing osteoclastic bone resorption during periods of elevated blood Ca levels [2,3]. Research utilizing non-equine models indicates that microbial metabolites have the potential to interact with host endocrine signaling pathways that regulate mineral metabolism. Nonetheless, there is an absence of direct evidence demonstrating the microbiota-mediated modulation of the parathyroid hormone (PTH), calcitriol, fibroblast growth factor 23 (FGF23), or calcitonin in equines. Consequently, the interactions between gut microbiota and the endocrine regulation of mineral homeostasis in horses should be regarded as hypothetical mechanisms rather than confirmed physiological pathways.

The regulation of mineral homeostasis by the endocrine system is intricately linked with the functions of the intestines, kidneys, and bones. These organs are in constant communication, exchanging hormonal and metabolic signals to sustain mineral equilibrium amidst varying nutritional and physiological states [1]. Recent research indicates that the gut microbiota might affect endocrine communication by changing the production of microbial metabolites, influencing mineral availability, and modulating epithelial and immune functions [10,12]. Although direct evidence in equines remains limited, these findings indicate that the interaction between endocrine regulation and gut microbial activity plays a critical role in mineral homeostasis.

### 4.4. Factors Affecting Mineral Bioavailability

The bioavailability of minerals is influenced by their dietary presence as well as various nutritional, physiological, and environmental factors that affect their intestinal absorption and utilization. The chemical form of a mineral plays a significant role, with highly soluble forms typically being absorbed more effectively than insoluble ones [17,97]. Dietary components may either enhance or impede mineral availability through synergistic or antagonistic interactions. Substances such as phytate, oxalate, tannins, and excessive dietary fiber can reduce mineral absorption by forming insoluble complexes with Ca, Fe, Zn, and other trace elements [98]. In contrast, organic acids, certain amino acids, and metabolites resulting from fermentation can improve mineral solubility and promote enhanced intestinal absorption [26,99]. Therefore, mineral bioavailability is contingent upon both the dietary makeup and the physicochemical environment within the gastrointestinal tract. In equines, the pronounced activity of hindgut microbial phytase appears to substantially mitigate the antinutritional effects of phytate commonly observed in monogastric species [100].

Host related factors significantly influence variations in mineral bioavailability. Factors such as age, physiological status, health, gastrointestinal function, and nutrient requirements play a crucial role in determining mineral absorption and retention [2,3]. Alterations in intestinal pH, epithelial integrity, transit time, and microbial fermentation modify the environment for mineral absorption. These changes influence mineral solubility, transporter activity, and epithelial function, thereby affecting mineral utilization. Increasing evidence suggests that the gut microbiota significantly contributes to these processes by shaping the intestinal microenvironment and producing metabolites that influence nutrient absorption [10,12]. Understanding these interactions is essential for interpreting the emerging role of the gut microbiota in mineral homeostasis, which will be examined in the subsequent section. Table 3 presents a summary of the primary host- and management-related factors that are known to affect the equine gut microbiota. Additionally, it outlines their plausible implications for mineral homeostasis, which are largely inferred rather than directly demonstrated.

## 5. Mechanisms Linking the Equine Gut Microbiota to Mineral Homeostasis

### 5.1. Remodeling of the Intestinal Microenvironment

The intestinal microenvironment functions as the primary interface through which the gut microbiota influences mineral homeostasis. Through microbial fermentation and other metabolic activities, the gut microbiota regulates factors such as luminal pH, oxygen tension, redox balance, mucus composition, epithelial integrity, and intestinal permeability [10,12,25]. These modifications impact the environment in which minerals are dissolved, transported, and absorbed by intestinal epithelial cells [103].

Microbial fermentation produces organic acids that can modify luminal pH, thereby altering the physicochemical environment where minerals are present. In other experimental systems, changes in luminal acidity have been linked to altered mineral solubility and availability [26,97]. The extent to which changes in hindgut pH mediated by short-chain fatty acids (SCFAs) directly influence systemic mineral absorption in horses remains uncertain. This uncertainty arises primarily because significant absorption of several minerals occurs prior to the main sites of hindgut fermentation. Furthermore, microbial metabolism is essential for preserving the integrity of the mucus layer and the intestinal epithelium. These structures act as the primary barrier between luminal contents and host tissues while also providing the surface necessary for mineral absorption [11,12]. Any disruption in epithelial integrity or microbial balance can potentially decrease mineral uptake by affecting both intestinal permeability and absorptive function.

Research in equine studies indicates that the gut microbiota plays a role in hindgut fermentation and the production of metabolites. Furthermore, evidence from other species suggests that microbial metabolites may also affect epithelial and mineral-related signaling pathways [13,14,62]. A robust intestinal barrier enhances nutrient transport efficiency while mitigating inflammation that could impair absorptive function. Conversely, microbial dysbiosis can compromise epithelial integrity, interfere with transporter activity, and reduce mineral bioavailability [104]. These findings collectively suggest that the gut microbiota creates a biological environment.

### 5.2. Microbial Metabolites as Regulators of Mineral Homeostasis

Microbial metabolites serve as the primary molecular mediators facilitating communication between the gut microbiota and the host. These compounds are synthesized during the fermentation of dietary substrates and through the microbial transformation of endogenous molecules. Once produced, they exert effects locally within the intestinal lumen or enter the circulation to influence distant organs [10,25].

SCFAs (acetate, propionate, and butyrate) are the predominant microbial metabolites generated during equine hindgut fermentation and are a major energy source for colonocytes, supplying an estimated 50–70% of the horse’s maintenance energy requirement [11,61]. Studies in humans and animals suggest that SCFA signaling might influence pathways related to mineral handling. However, there is limited direct evidence connecting SCFA production to changes in mineral absorption or transporter activity in horses. Thus, SCFAs should be considered as potential mediators of microbiota–host communication related to mineral balance, rather than confirmed regulators of mineral absorption in horses.

The gut microbiota produces a range of metabolites, encompass secondary bile acids, indole derivatives, polyamines, microbial vitamins, and other bioactive compounds generated during microbial metabolism [25,27]. Although the majority of these metabolites have predominantly been investigated in humans and laboratory animals, they are increasingly acknowledged as regulators of epithelial renewal, immune function, oxidative balance, and cellular signaling. Moreover, certain metabolites affect the expression and activity of intestinal transport proteins, suggesting a possible indirect involvement in mineral absorption [12]. However, the precise contributions of these metabolites to mineral homeostasis in horses are not well-understood, warranting additional investigation. These metabolites function through various interconnected mechanisms, rather than a single pathway, to collectively influence the efficiency of mineral absorption and utilization. Understanding these mechanisms is essential for examining how the gut microbiota affects the homeostasis of specific minerals.

### 5.3. Microbial Regulation of Intestinal Mineral Transport

Efficient mineral absorption relies on the coordinated activity of intestinal epithelial transport systems that respond to dietary, physiological, hormonal, and environmental signals [10,12]. Experimental studies in humans and other animal models indicate that microbial metabolites can influence signaling pathways associated with intestinal mineral transport. However, direct experimental evidence showing that the equine gut microbiota or its metabolites regulate mineral transporter expression or activity in horses remains limited [27]. For instance, the effects of short-chain fatty acids (SCFAs) on calcium transport proteins, including TRPV6 and calbindin-D9k, have been demonstrated in both human and rodent models. In contrast, alterations in iron regulatory pathways associated with microbiota, involving DMT1, ferroportin, and hepcidin, have primarily been examined in non-equine systems. Similarly, evidence regarding zinc- and copper-associated transport pathways is largely obtained from non-equine models. Accordingly, the mechanisms discussed in this section should be interpreted according to their evidentiary basis, with established transporter physiology distinguished from cross-species evidence and proposed microbiota-mediated effects.

The intestinal epithelium functions as a dynamic interface between the gut microbiota and the host. The ongoing interaction between microbial communities and epithelial cells modulates cell proliferation, differentiation, and renewal, thus maintaining absorptive capacity across the gastrointestinal tract [12,105]. In cases of intestinal dysbiosis, disruptions in regulatory processes can result in decreased transporter activity [103]. Therefore, sustaining a balanced gut microbiota is essential for maintaining the physiological conditions required for effective mineral transport.

While the fundamental mechanisms of intestinal mineral transport share similarities, each mineral possesses unique absorption pathways, regulatory hormones, and sensitivity to microbial activity. Consequently, the influence of the gut microbiota on minerals differs according to their chemical properties, transport systems, and physiological regulation. The following sections summarize the current evidence linking the gut microbiota to the homeostasis of specific macro- and trace minerals.

### 5.4. Gut Microbiota and Macro Mineral Homeostasis

Ca, P, and Mg are the primary macro minerals essential for skeletal development, neuromuscular function, cellular signaling, and energy metabolism. The homeostasis of these minerals is maintained through intricate interactions among the intestine, bone, kidneys, and endocrine system [1,4,20]. Recent evidence suggests that the gut microbiota constitutes an additional element of this regulatory network by altering the intestinal environment where these minerals are absorbed [1]. This perspective expands the traditional model of mineral homeostasis by recognizing the intestinal microbiota as an active participant in mineral regulation, rather than merely a passive component of digestion, prior to endocrine regulation.

Among the macro minerals, Ca absorption is most directly shaped by the fermentation-driven pH and epithelial barrier mechanisms described in Section 5.1. In experimental studies involving rodent colorectal mucosa and Caco-2 human intestinal epithelial cells, exposure to short-chain fatty acids (SCFAs) has been demonstrated to enhance the expression of TRPV6 and calbindin-D9k [20]. These findings suggest a potential molecular mechanism linking microbial activity with calcium handling; however, the specific microbiota–TRPV6/calbindin relationship has not been directly demonstrated in horses.

The influence of gut microbiota on P and Mg homeostasis has not been as extensively investigated as its effect on Ca; however, it is increasingly being acknowledged. Microbial phytase activity can improve P availability by hydrolyzing phytate-bound phosphorus, thereby augmenting the pool of absorbable inorganic phosphate [86]. Additionally, microbial fermentation creates physicochemical conditions that favor Mg solubility and intestinal absorption, although the exact molecular mechanisms have not yet been fully elucidated [21,26]. Ca, P, and Mg utilize shared absorptive pathways and are regulated by common endocrine mechanisms. Consequently, alterations in gut microbial activity may impact the collective balance of these minerals rather than affecting each mineral independently. This integrated regulation highlights the importance of macro mineral homeostasis as a coordinated physiological process.

Although there is an increasing amount of mechanistic evidence, the majority of the current understanding is based on human studies and experimental animal models. Research on similar topics in equines remains limited, especially concerning the microbial regulation of mineral transporters, endocrine signaling, and long-term mineral metabolism. Future studies integrating metagenomics, metabolomics, and mineral balance analyses are essential to clarify these interactions within practical equine feeding contexts. These investigations will establish a stronger scientific basis for developing microbiome-targeted nutritional strategies to improve mineral utilization and skeletal health in equines. Table 4 provides a summary of the principal microbial groups identified within the equine gastrointestinal tract, highlighting their primary sites of activity, predominant metabolites, and potential implications for mineral homeostasis.

### 5.5. Gut Microbiota and Trace Mineral Homeostasis

Trace minerals play a vital role in oxygen transport, antioxidant defense, immune regulation, enzyme activity, and cellular metabolism, albeit in relatively small quantities. The regulation of the intestinal absorption of trace minerals is highly stringent due to the potential negative impact of both deficiency and excess on physiological functions [2,3]. In contrast to macro-minerals, trace minerals engage in direct interactions with the gut microbiota. These microorganisms compete with the host for specific trace elements, alter their chemical forms, and affect the expression of epithelial transport proteins that facilitate mineral uptake [27,106]. The interaction between gut microbiota and trace mineral homeostasis is highly dynamic, extending beyond simple nutrient absorption.

Fe, a trace mineral, has been the subject of extensive research concerning gut microbiota. It plays a critical role in oxygen transport, mitochondrial respiration, and various enzymatic processes. Concurrently, Fe is essential for the proliferation of numerous intestinal microorganisms, resulting in competition between the host and microbiota for this limited resource [87,107]. Research involving both human subjects and experimental animal models suggests that the composition of gut microbiota and the metabolites they produce can affect iron availability, inflammatory signaling, and pathways associated with DMT1, ferroportin, and hepcidin [88]. However, direct evidence of the microbiota-mediated regulation of DMT1, ferroportin, or hepcidin in horses is currently lacking. Therefore, these findings should be considered cross-species mechanistic evidence that provides hypotheses for equine research rather than established equine mechanisms [87].

Zn and Cu serve as crucial cofactors for various enzymes that participate in antioxidant defense, immune function, and cellular metabolism. Their absorption is mediated by specialized epithelial transport systems and is affected by the condition of the intestinal barrier. Research conducted on non-equine models indicates that microbial metabolites, along with alterations in gut microbial composition, may affect epithelial integrity and the signaling pathways involved in zinc and copper regulation [27,89]. Whether these interactions directly regulate the expression of Zn or Cu transporters or the absorption of minerals in horses remains unresolved. Interactions between Zn, Cu, and other trace minerals may influence their intestinal availability [106]. However, direct evidence detailing these mechanisms in horses is limited.

Unlike the well-documented interactions of Fe, Zn, and Cu with gut microbiota, the relationships between gut microbiota and the homeostasis of Se and Mg remain less comprehensively understood. Se is incorporated into selenoproteins, which serve to protect cells from oxidative damage and modulate immune function. In contrast, Mg functions as a cofactor for enzymes that are crucial for bone development, antioxidant defense, and energy metabolism [91,93]. Recent evidence suggests that intestinal microorganisms may affect Se metabolism through the microbial transformation of Se compounds, and indirect effects on Mg absorption have also been suggested [27,92].

Current evidence collectively suggests that the gut microbiota affect trace mineral homeostasis through various complementary mechanisms. These mechanisms encompass competition for minerals, transformation of mineral compounds, and maintenance of intestinal barrier function. Nevertheless, the strength of the evidence varies significantly among individual trace minerals and is predominantly based on studies conducted in humans and experimental animal models. Expanding mechanistic research in horses is therefore essential to determine the physiological significance of these interactions and their implications for equine nutrition. Table 5 provides a summary of the current evidence regarding the relationship between gut microbiota and trace mineral homeostasis in horses, detailing the availability of direct equine data for each mineral.

### 5.6. Integrated Model of Gut Microbiota–Mineral Homeostasis

This review supports a conceptual model in which the gut microbiota may contribute to mineral homeostasis through an interconnected network of microbial, physicochemical, epithelial, immune, and endocrine pathways. The strength of evidence among mechanisms varies significantly, with many proposed molecular interactions being primarily supported by studies conducted on humans or experimental animal models, rather than direct equine experiments. These processes function concurrently and interact with the classical intestine, bone, kidney, and endocrine axis to maintain systemic mineral balance. Consequently, the gut microbiota may constitute an additional element within the mineral-homeostasis network, complementing rather than replacing the established intestinal, skeletal, renal, and endocrine mechanisms.

**Figure 4:**
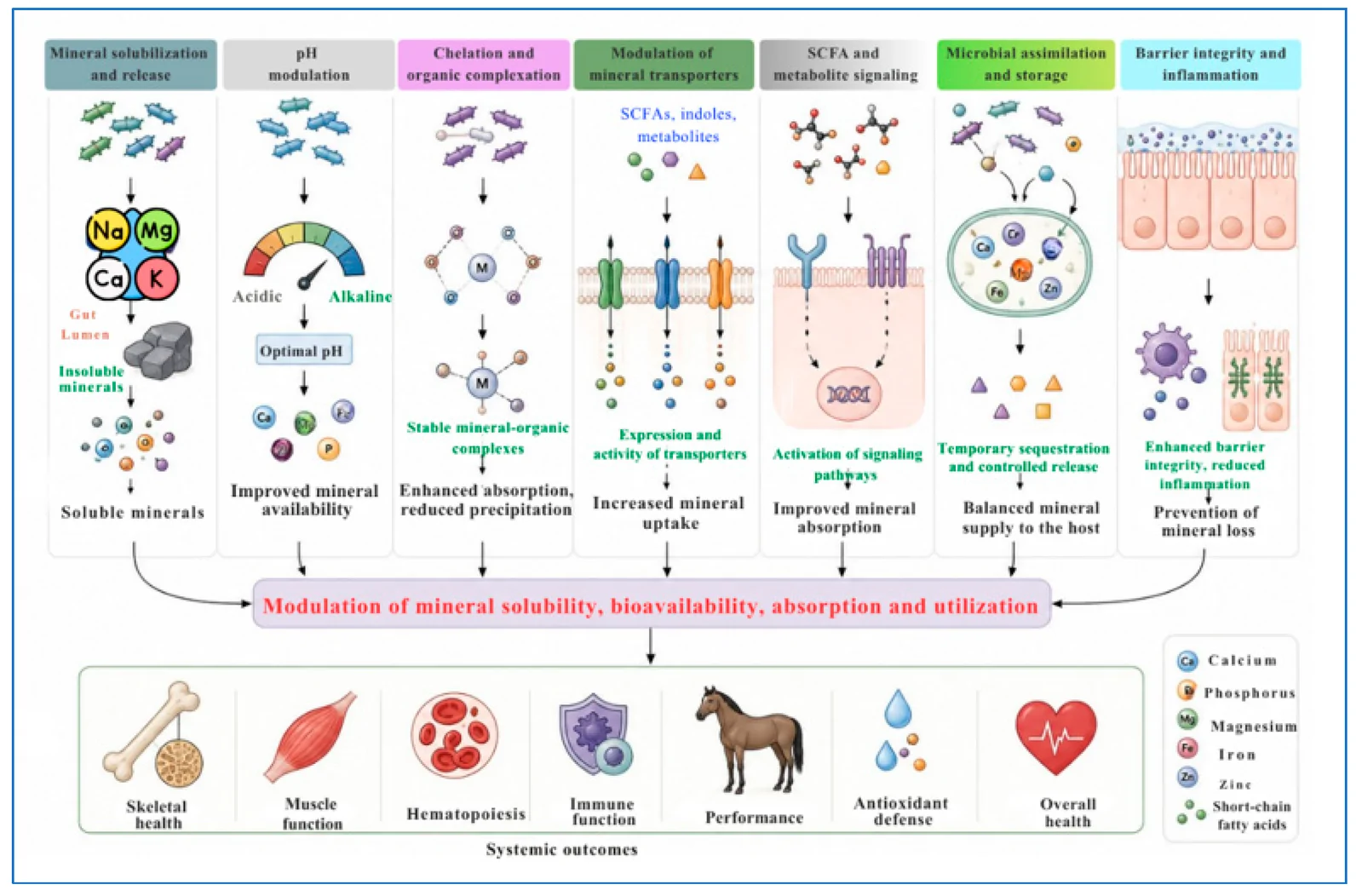
Integrated mechanisms through which the gut microbiota regulates mineral homeostasis in horses. Microbial metabolism and microbial-derived metabolites interact with the intestinal environment, epithelial barrier, immune system, and mineral transport processes to influence mineral solubility, bioavailability, absorption, transport, and systemic utilization. These interconnected pathways provide a conceptual framework linking the equine gut microbiota with mineral homeostasis and physiological health. Created by authors using BioRender. https://BioRender.com/t6qp6d4 (accessed on 12 August 2026).

The interactions between the gut microbiota and mineral homeostasis are both dynamic and bidirectional. Dietary composition influences both microbial diversity and metabolic activity, while microbial metabolites alter the intestinal environment and affect mineral availability. Changes in mineral status can also impact the structure of microbial communities, establishing a reciprocal feedback loop between host nutrition and the intestinal microbiome [27]. Consequently, mineral homeostasis should be considered the result of ongoing interactions among diet, gut microbiota, intestinal physiology, endocrine regulation, and host metabolism, rather than the function of any single organ or pathway. Figure 4 provides a summary of the integrated pathways underlying these interactions.

### 5.7. Direct Evidence from Equine Studies

While most mechanistic evidence discussed above derives from human and rodent studies, a small but growing body of direct equine research provides an initial, genuinely species-specific foundation for this framework. Metagenomics sequencing of equine fecal samples has advanced the understanding of the functional gene content within the hindgut microbiome, surpassing mere taxonomic profiling. This approach has enabled the recovery of over a hundred metagenome assembled bacterial and archaeal genomes, thereby uncovering extensive and largely uncharacterized functional diversity [59]. On a broader scale, the construction of an integrated gut microbial gene catalog from elite endurance horses, which includes over 25 million non-redundant genes, has demonstrated the feasibility of holoomic approaches. These approaches, which integrate microbiome and host metabolomics data, have successfully linked microbial gene function to host mitochondrial and metabolic phenotypes [110]. Although this catalog was applied to cardiovascular fitness rather than mineral metabolism, it establishes the methodological precedent and reference dataset that mineral focused equine microbiome studies could build upon.

Direct evidence is also available regarding mineral status itself. Serum concentrations of Cu, Zn, Fe, Ca, K, and Mg have been assessed in horses under various physiological and antioxidant supplementation conditions. These assessments demonstrate that the status of equine trace and macro minerals is measurably influenced by diet and exercise [111]. Additionally, the significant hindgut microbial phytase activity observed in horses Brinkley-Bissinger et al. [100] provides a mechanistically specific, equine-validated connection between the gut microbiota and P bioavailability, rather than relying on extrapolations from other species. While these studies are primarily descriptive compared to the mechanistic insights obtained from human and rodent models, they do not establish a direct connection between microbiome composition or function and the expression of mineral transporters or endocrine regulation of minerals in horses. However, they indicate that the necessary metagenomic, metabolomic, and mineral-analytical methodologies to address this gap are already accessible and validated within equine research.

## 6. Future Research Priorities and Translational Perspectives

Despite significant progress in microbiome research, the interactions between gut microbiota and mineral homeostasis in horses remain inadequately understood. Existing studies primarily focus on characterizing microbial composition rather than clarifying the molecular mechanisms through which intestinal microorganisms influence mineral absorption, transport, and systemic metabolism [108]. Moreover, much of the mechanistic evidence has been derived from human and laboratory animal models, limiting its applicability to equine physiology. Addressing these knowledge gaps requires transitioning from descriptive microbiome studies to hypothesis-driven mechanistic investigations specifically designed for equine subjects. The issue of sampling remains unresolved in the literature. Although the current analysis aligns with evidence suggesting divergence between fecal and intestinal microbiota signals [112,113], other studies have indicated that fecal bacterial communities do not significantly differ from those in the colon or caecum, but only from the upper gastrointestinal tract [110,114,115]. This discrepancy underscores the necessity for direct comparative studies that specifically address mineral-relevant taxa across different gut regions.

Future research should incorporate controlled nutritional interventions in conjunction with comprehensive physiological and molecular analyses to establish causal relationships between the gut microbiota and mineral homeostasis. By integrating mineral balance studies with microbiome profiling, microbial metabolite analysis, intestinal transporter expression, endocrine responses, and immune biomarkers, our understanding of host–microbiota interactions will be enhanced. The application of metagenomics, meta-transcriptomic, metabolomics, and host transcriptomics will further facilitate the identification of microbial pathways and signaling networks involved in mineral metabolism under varying physiological and nutritional conditions. Evidence supporting this experimental approach is already available in equine studies. A recent in vitro batch fermentation study, utilizing a SHIME-derived colon-simulation system, demonstrated that the equine hindgut microbiota are capable of degrading a small toxic plant-derived amino acid. This was accompanied by the quantification of SCFA and shifts in the 16S rRNA community [116]. By adapting this experimental framework to replace the toxin with dietary minerals or phytate, a controlled, host-independent system could be established to directly test the mechanistic hypotheses previously proposed.

In future research on equine mineral–microbiota interactions, it is essential to carefully consider study design and sampling methodology. Comparative studies in other species have demonstrated that fecal and intestinal samples produce distinct microbiome signals. Specifically, fecal samples tend to more accurately reflect the host’s diet, whereas intestinal samples capture unique regional microhabitat effects [112]. This distinction is particularly pertinent to mineral-homeostasis research, as luminal mineral concentrations and transporter expression exhibit significant variation along the equine gastrointestinal tract. Consequently, exclusive reliance on fecal sampling may inadequately represent region-specific interactions between microbiota and minerals. A recent study involving cecal cannulation in horses directly confirmed that fecal samples do not accurately reflect cecal pH, buffering capacity, or microbial composition across different dietary treatments [113]. This finding underscores the necessity of employing intestinally invasive or cannulated sampling methods to accurately characterize microbiota–mineral relationships at the absorption site. Longitudinal data were essential for this study. The first comprehensive 12-month survey of the fecal microbiota in healthy pastured horses revealed that forage supplementation, seasonal variations, and ambient weather significantly influenced compositional changes, even in clinically normal animals [46]. This finding highlights the necessity of considering these variables in the design of mineral balance studies. Furthermore, meta-analyses of gut microbiome studies in human diseases indicate that certain conditions are characterized by an increase in potentially pathogenic taxa, while others show a depletion of health-associated bacteria [117]. Whether a similar pattern exists in mineral-related disorders in horses remains unexplored and presents an opportunity for future research.

An enhanced mechanistic understanding will facilitate the development of microbiome informed nutritional strategies aimed at improving mineral utilization and gastrointestinal health. Rather than simply augmenting dietary mineral concentrations, future nutritional approaches may focus on optimizing the intestinal microbial ecosystem to enhance mineral bioavailability and absorption. The composition of dietary fiber, along with prebiotics, probiotics, synbiotics, postbiotics, and fermentation modulating feed additives, represents promising tools for manipulating the gut microbiota. These strategies aim to support mineral homeostasis while reducing unnecessary mineral supplementation and environmental mineral excretion.

In addition to mineral nutrition, a microbiome informed strategy may enhance skeletal development, immune competence, reproductive efficiency, and athletic performance throughout the various life stages of horses, ranging from growing foals to geriatric animals. To fully realize this potential, ongoing interdisciplinary research is necessary. This research should integrate nutrition, microbiology, physiology, molecular biology, and systems biology to convert mechanistic discoveries into evidence-based and sustainable feeding practices.

Future advancements in equine microbiome research are anticipated to increasingly rely on the integration of multi-omics technologies. These technologies are capable of concurrently characterizing microbial composition, gene expression, metabolic activity, protein synthesis, and metabolite production. The combined use of shotgun metagenomics, metatranscriptomics, metabolomics, proteomics, and systems biology approaches will facilitate the functional characterization of microbial pathways involved in nutrient metabolism and host physiology, extending beyond taxonomic profiling. Such integrative methodologies are projected to support precision nutrition strategies and microbiome-informed interventions aimed at enhancing mineral utilization and equine health [118,119].

## 7. Conclusions

Mineral homeostasis in horses is influenced by a complex interaction involving intestinal absorption, systemic regulation, and tissue and renal processing, with the gut microbiota emerging as a potential additional factor in this process. Microbial fermentation, metabolite production, and alterations in the intestinal environment offer plausible mechanisms through which microbial activity may affect mineral availability and host physiology. Nonetheless, direct evidence in equines is limited, particularly concerning the causal relationships between specific microbial taxa or metabolites and systemic mineral regulation. Several proposed molecular mechanisms, such as effects on calcium transport pathways and interactions with DMT1, ferroportin, hepcidin, and zinc- and copper-associated transport systems, have primarily been supported by studies in humans and other experimental models. Although these findings suggest plausible mechanisms in horses, they cannot be regarded as established equine pathways. A significant research gap exists because of the lack of controlled equine studies directly linking gut microbial activity with mineral absorption, transporter function, and systemic mineral balance.

## Figures and Tables

**Table 1:** Major microbial groups inhabiting the equine hindgut, their ecological roles, metabolic functions, and potential implications for mineral homeostasis.

Microbial Group	Genera	Relative Abundance	Primary Site of Activity	Ecological Role	Major Metabolites	Potential Implications for Mineral Homeostasis	References
*Firmicutes*	*Ruminococcus*, *Faecalibacterium*, *Roseburia*, *Lachnospiraceae*	Dominant	Cecum and large colon	Primary fibrolytic fermenters causing the degradation of structural carbohydrates	Butyrate, acetate	Butyrate may influence epithelial barrier integrity, lowers luminal pH, and direct effects on mineral absorption in horses remain unconfirmed	[9,11,33,36]
*Bacteroidota *(*Bacteroidetes*)	*Prevotella*, *Bacteroides*, *Rikenellaceae*	Dominant	Cecum and large colon	Degradation of hemicellulose, pectin, resistant starch, and proteins	Propionate, acetate	Butyrate strengthens barriers, lowers luminal pH, and may improve mineral absorption in horses	[8,35,37,49]
*Fibrobacterota*	*Fibrobacter succinogenes*	Low abundance	Cecum	Cellulolytic bacteria that efficiently degrade crystalline cellulose	Succinate, acetate	Produces substrates that facilitate microbial cross-feeding, maintaining fiber fermentation and the release of nutrients	[30,33,37]
*Verrucomicrobiota*	*Akkermansia* spp.	Low abundance	Mucus layer of cecum and colon	Mucin degradation and maintenance of mucus turnover	Acetate, propionate	Maintains the integrity of epithelial tissues and the balance of mucosal environments, which may indirectly aid in the absorption of nutrients and minerals	[37,50,51]
*Proteobacteria*	*Escherichia*, *Succinivibrio*, *Desulfovibrio*	Minor	Throughout the intestine (higher in proximal regions)	Facultative anaerobes to environmental changes	Organic acids	Dysbiosis, inflammation of the intestines, and reduced nutrient absorption are often linked to expansion	[8,35,47]
Methanogenic archaea	*Methanobrevibacter*, *Methanocorpusculum*	Rare	Cecum and large colon	Hydrogen scavengers supporting anaerobic fermentation	Methane	Maintaining fermentation efficiency and sustaining SCFA production are achieved by removing hydrogen	[41,52]
Anaerobic fungi	*Piromyces*, *Neocallimastix*, *Caecomyces*	Present	Fiber particles within the hindgut	Enzymatic disruption of lignocellulosic plant material through carbohydrate-active enzymes (CAZymes)	Acetate, formate, H_2_, ethanol	By improving the availability of substrates to fibrolytic bacteria, fiber digestion and fermentation become more efficient	[7,53,54]
Ciliate protozoa	*Blepharocorys*, *Bundleia*, *Cycloposthium*	Present	Cecum and colon	Fiber and starch utilization, bacterial predation, and regulation of microbial turnover	SCFAs and fermentation intermediates	Play a role in maintaining the stability of the microbial ecosystem and facilitating nutrient cycling in the hindgut	[55]
Bacteriophages (Virome)	Siphoviridae-, Podoviridae-, and Myoviridae-like dsDNA phages	Emerging	Hindgut microbiome	Regulation of bacterial populations, horizontal gene transfer	—	Microbial community structure is shaped, ecosystem resilience is influenced, and there may be indirect effects on nutrient metabolism and mineral homeostasis	[41,56,57]

**Table 2:** Major minerals involved in equine physiology, their biological functions, regulatory mechanisms, and potential interactions with the gut microbiota.

Mineral	Classification	Major Physiological Functions	Primary Absorption Site	Principal Regulatory Mechanisms	Potential Interactions with the Gut Microbiota	Evidence Level	References
Ca	Macro mineral	Skeletal mineralization, muscle contraction, nerve transmission, intracellular signaling	Duodenum and jejunum	PTH, calcitriol (1,25-dihydroxyvitamin D_3_), calcitonin	Alterations in luminal chemistry associated with short-chain fatty acids (SCFAs) may affect calcium solubility; however, direct enhancement of calcium absorption in horses has not been confirmed.	Cross-species/proposed	[2,3,10,12,20]
P	Macro mineral	Bone mineralization, ATP synthesis, nucleic acids, phospholipids	Jejunum	PTH, FGF23, calcitriol	Microbial phytase activity and fermentation may influence phosphorus availability	-	[1,3,22,86]
Mg	Macro mineral	Enzyme activation, neuromuscular function, energy metabolism	Small intestine and large intestine	Intestinal absorption, renal regulation, vitamin D	SCFAs mediated changes in luminal conditions and epithelial function may facilitate magnesium absorption	Proposed	[3,10,21,27,87,88]
Fe	Trace mineral	Oxygen transport, erythropoiesis, mitochondrial respiration	Duodenum	Hepcidin, ferroportin	Microbial metabolites have been implicated in iron regulatory pathways in non-equine models; however, direct microbiota-mediated regulation of equine iron transport remains unconfirmed.	Cross-species	
Zn	Trace mineral	Enzyme activity, immune function, antioxidant defense	Small intestine	ZIP and ZnT transporters	Changes in epithelial function associated with microbiota may influence zinc availability and transporter pathways; however, direct evidence in equines is limited.	Cross-species/proposed	[27,89]
**Cu**	Trace mineral	Connective tissue formation, antioxidant enzymes	Small intestine	ATP7A and ATP7B transporters	Changes in intestinal physiology associated with microbiota may influence copper handling; however, direct mechanistic evidence in horses is lacking.	Proposed	[3,90]
Se	Trace mineral	Antioxidant defense, thyroid hormone metabolism, immune regulation	Small intestine	Selenoprotein synthesis	Gut microorganisms participate in selenium metabolism and may influence its bioavailability	-	[27,91,92]
Mn	Trace mineral	Bone development, cartilage formation, carbohydrate metabolism	Small intestine	DMT1 and related transport systems	Microbial fermentation may indirectly influence manganese absorption by modifying the intestinal environment.	-	[3,93]

PTH: Parathyroid hormone, FGF23: Fibroblast growth factor 23, SCFAs: Short-chain fatty acids, DMT1: Divalent metal transporter 1.

**Table 3:** Major factors influencing the equine gut microbiota and their potential implications for mineral homeostasis.

Factor	Effect on Gut Microbiota	Possible Implication for Mineral Homeostasis	References
Diet	Alters microbial diversity and SCFA production	Changes mineral solubility and absorption	[7,30,45]
Antibiotics	Reduces diversity; induces dysbiosis	May impair mineral bioavailability	[16]
Age	Shapes microbial maturation	Influences mineral requirements and utilization	[34]
Exercise	Alters microbial metabolism	May affect antioxidant mineral metabolism	[101]
Transportation/Stress	Modifies microbial composition	Potentially alters intestinal barrier function	[102]
Disease	Causes dysbiosis	Disturbs mineral homeostasis	[8]

**Table 4:** Microbiota-mediated mechanisms influencing macro mineral homeostasis in horses.

Microbial Mechanism	Primary Biological Action	Potential Impact on Mineral Homeostasis	Evidence in Horses	Overall Evidence	References
SCFA production	Lowers luminal pH; acts as a signaling molecule	May alter mineral solubility and epithelial conditions relevant to absorption; direct equine evidence is limited	Limited	Strong (non-equine)	[10,26]
Organic acid production	Acidifies the intestinal lumen	May alter mineral dissolution and bioavailability	Limited	Moderate	[12,13,97]
Maintenance of epithelial integrity	Strengthens tight junctions and mucus layer	May support epithelial conditions required for nutrient and mineral transport	Limited	Strong	
Regulation of epithelial transporters	Alters transporter expression and activity	May regulate active transport of calcium, phosphorus, and magnesium	No direct evidence	Emerging	[20]
Microbial phytase activity	Hydrolyzes phytate-bound phosphorus	May increase phosphorus availability for intestinal absorption	Limited	Moderate	[86]
Microbiota–endocrine interactions	Modulates vitamin D, PTH, and FGF23 signaling	May indirectly regulate systemic macro mineral homeostasis	No direct evidence	Emerging	[1,4]

**Table 5:** Current evidence linking the gut microbiota with trace mineral homeostasis in horses.

Trace Mineral	Major Physiological Functions	Microbiota-Mediated Mechanisms	Potential Effects on Mineral Homeostasis	Evidence in Horses	Strength of Mechanistic Evidence	References
Iron (Fe)	Oxygen transport, mitochondrial respiration, enzyme activity	Competition for iron; regulation of DMT1, ferroportin, and hepcidin; modulation of intestinal inflammation	Alters iron bioavailability, intestinal absorption, and systemic iron metabolism	Physiological data available; direct microbiota-mediated mechanistic evidence lacking	Cross-species mechanistic evidence	[87,88,107]
Zinc (Zn)	Antioxidant defense, immune regulation, epithelial integrity	Regulation of ZIP/ZnT transporters, epithelial barrier integrity, and microbial metabolite signaling	Influences zinc absorption, epithelial repair, and immune homeostasis	Direct microbiota–transporter evidence in horses lacking; mechanistic evidence primarily from other species	Strong	[27,89,106]
Copper (Cu)	Connective tissue formation, antioxidant enzyme activity, iron metabolism	Modulation of ATP7A/ATP7B transporters and interactions with iron and zinc metabolism	May influence copper absorption, antioxidant capacity, and trace mineral balance	Observational evidence only; direct mechanistic evidence in horses lacking	Emerging	[27,90,91,106]
Selenium (Se)	Antioxidant defense, thyroid hormone metabolism, immune regulation	Microbial biotransformation of selenium compounds and regulation of selenoprotein metabolism	May modify selenium bioavailability, antioxidant capacity, and immune function	Observational evidence only; limited equine evidence; microbial transformation mechanisms primarily inferred from other systems	Moderate	[27,91,92,108,109]
Manganese (Mn)	Bone development, cartilage formation, enzyme activation	Proposed indirect effects through microbial fermentation and alterations in intestinal physiology	Possible influence on manganese absorption and utilization	No direct equine microbiota-mineral evidence identified	Hypothetical	[27,93]

DMT1: Divalent metal transporter 1, ZIP/ZnT: Zinc importer (ZIP) and zinc transporter (ZnT) protein families, ATP7A/ATP7B: Copper-transporting ATPases.

## Data Availability

No new data were created or analyzed in this study. Data sharing is not applicable to this article.
